# The Prevalence of Activities of Daily Living Impairment in Patients With Heart Failure: A Systematic Review and Meta-Analysis

**DOI:** 10.3389/fcvm.2022.952926

**Published:** 2022-07-14

**Authors:** Jing Lu, Meng Wang, Yue Zhang, Lifen Mao, Xiaoxiao Chen, Rulan Yin, Xiaoqing Shi

**Affiliations:** ^1^Department of Nursing, The First Affiliated Hospital of Soochow University, Suzhou, China; ^2^School of Nursing, Suzhou Medical College of Soochow University, Suzhou, China; ^3^Department of Rheumatology, The First Affiliated Hospital of Soochow University, Suzhou, China; ^4^Faculty of Nursing, Chiang Mai University, Chiangmai, Thailand

**Keywords:** prevalence, activities of daily living, impairment, heart failure, meta-analysis

## Abstract

**Objectives:**

The prevalence of activities of daily living (ADL) in patients with heart failure (HF) reported in current studies were inconsistent, ranging from 11.1 to 70.5%. The purpose of this study is to quantify the prevalence of ADL impairment in HF patients.

**Methods:**

PubMed, Embase, Cochrane, CINAHL, CNKI, SinoMed, VIP, and Wanfang databases were systematically searched for relevant studies (up to March 2, 2022). Cross-sectional, case-control, or cohort studies with detailed descriptions of overall ADL impairment in HF were included. Stata 16.0 was used for statistical analysis. Fixed-effect or random-effect model was adopted according to heterogeneity which was evaluated by Cochran’s *Q* and *I*^2^ values. Sensitivity analysis, subgroup analysis, and meta-regression were performed to investigate the sources of heterogeneity.

**Results:**

A total of 12 studies with 15,795 HF patients were included in the meta-analysis, and the pooled prevalence of ADL impairment in patients with HF was 38.8% (95%CI: 28.2–49.3%; *I*^2^ = 99.5%, *P* < 0.001). No possible sources of heterogeneity were found in subgroup analysis and meta-regression. Funnel plots and Egger’s test showed no publication bias (*P* = 0.595).

**Conclusion:**

The prevalence of ADL impairment is relatively high in HF patients. Differences in the prevalence of ADL impairment in patients with HF may be influenced by country, region, and assessment time. We suggest that more researchers could focus on the changes of ADL impairment in HF patients during different disease periods in different regions and countries.

## Introduction

Heart failure (HF) is a complex clinical syndrome characterized by deterioration of the heart and other systems such as the kidneys, liver, lungs, and muscles. It is estimated that 64.3 million people worldwide suffer from HF (1). The HF prevalence rises with age (2). More than 75% of hospitalization related to HF occurred in those aged 65 and older (3). The main clinical features of HF are fatigue, dyspnea, lower extremity edema, cough, precordial pain, dizziness, and palpitations. In the elderly, dyspnea and fatigue are prominent, which may lead to exercise intolerance and eventually to dependence on activities of daily living (ADL) (4, 5). In addition, HF is known to alter the function of skeletal muscle contraction units. Individuals with this chronic disease typically suffer from muscle weakness, which reduces their level of physical activity and their ability to maintain balance (6), leading to the patient’s inability to perform ADL independently (such as transferring, bathing, and toileting). As one of the most fundamental ability, ADL refers to the repetitive primary acts that people must accomplish in their daily life in order to meet their basic needs. It is a measurement tool for body objective condition, as well as an indication of health. ADL ability in HF patients is closely related to their prognosis. A study showed that patients with ADL impairment had an approximately threefold increase in 3-month readmission rates compared to patients without ADL impairment (7). Another study showed that ADL ability was an independent predictor of all-cause mortality in patients with HF (8). Physical limitations and loss of independence can complicate patients’ care and reduce patients’ quality of life (9). Therefore, it is crucial to adopt reasonable interventions to improve the ADL ability of HF patients.

To allow a more rational allocation of medical resources, the prevalence of ADL impairment among patients with HF must be identified first. However, currently, investigations on this topic give a wide range of results. In a study from America (US), the ADL impairment prevalence was 11.1% (10). One study from Europe (Poland) showed that the prevalence of ADL impairment was 26.3% (11). However, ADL impairment prevalence in an Asia (Japan) study was 70.5% (12). Those showed great differences between different regions and countries, and no systematic review and meta-analysis has been found. Hence, the purpose of this study is to quantify the prevalence of ADL impairment in patients with HF, so as to provide a basis for medical decision making in the management of HF patients.

## Methods

This systematic review and meta-analysis was based on the preferred report items in the Preferred Reporting Items for Systemic Reviews and Meta-Analyses (PRISMA 2020) guidelines.

### Search Strategy

We searched four English databases (PubMed, Embase, Cochrane and CINAHL) and four Chinese databases (CNKI, Wanfang, VIP and SinoMed) for studies related to ADL impairment in patients with HF (from inception to March 2, 2022). We used the following search terms, adapted for each database when appropriate: ADL (“activities of daily living” or “ADL” or “Daily Living Activities” or “Chronic Limitation of Activity” or “ADL disability”) and HF (“heart failure” or “cardiac failure” or “cardiac dysfunction” or “cardiac insufficiency” or “Myocardial failure” or “Heart Decompensation” or “Congestive Heart Failure”) (the full list of search terms is provided in Supplementary File 1). We also reviewed the list of references in the included studies to obtain additional studies.

### Inclusion/Exclusion Criteria

Inclusion criteria were: (1) Patients were diagnosed with HF; (2) The study included a detailed description of the assessment scale of ADL; (3) Complete baseline data of the total prevalence of ADL impairment was provided; (4) The study design was cross-sectional, case-control, or cohort; and (5) Research published in English or Chinese.

Exclusion criteria were: (1) meeting abstract, case report, review, meta-analysis, letter, pilot study or qualitative study; or (2) duplicate studies and/or data (When there are different studies in the same unit and the same sample, the most recent one is selected).

Two reviewers screened the literature separately according to the inclusion and exclusion criteria, and a third reviewer made a judgment if there were conflicts.

### Data Extraction and Quality Assessment

Two investigators retrieved data from eligible studies independently, while a third investigator double-checked for accuracy. Data extracted included the first author, publication time, country, design, sample size, sex, age, type of patient, NYHA class III-V, LVEF, assessment scale, assessment time (Sources), and ADL impairment (cut-off, prevalence).

Quality of the included studies was evaluated using the modified Newcastle-Ottawa Scale (M-NOS) (12). There are 5 items, with 1 point given for each “Yes” answer and 0 point given for each “No” answer. The overall score runs from 0 and 5, with higher scores signifying higher quality. In this study, ≥ 3 was defined as low-risk bias and < 3 as high-risk bias.

### Data Analysis

For statistical analysis, Stata 16.0 was employed. To examine heterogeneity, Cochran’s chi-square test (Cochran’s *Q*-value) and *I*^2^-value were used, with *P* < 0.05 or *I*^2^ > 50% indicating significant heterogeneity between studies. Fixed-effect model was performed to calculate the pooled prevalence of ADL impairment in patients with HF when there was no significant heterogeneity, and random-effect model was used otherwise. Subgroup analysis, sensitivity analysis and meta-regression analysis were undertaken to identify the sources of heterogeneity. Funnel plots and Egger’s test were conducted to evaluate publication bias.

## Results

### Study Selection

**FIGURE 1:**
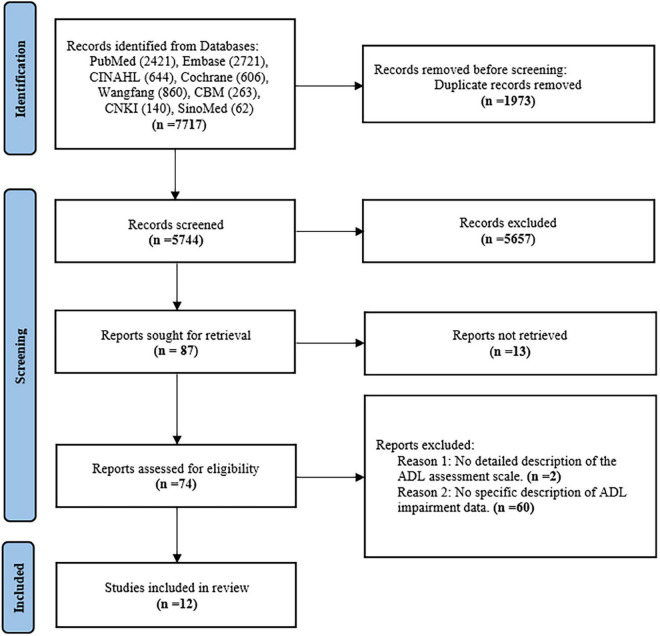
Flow-chart illustrating the article search process.

A total of 12 studies were included in this systematic review and meta-analysis, involving 15,795 patients with HF. Sample size of the included studies ranged from 180 to 4,735. Details were showed in Figure 1.

### Basic Information of the Included Studies

In this review, 5 studies were occurred in America (10, 13–16), 4 in Asia (7, 8, 12, 17) and 3 in Europe (11, 18, 19). The prevalence of ADL impairment in HF patients ranged from 11.1 to 70.5%. All studies received ≥ 3 points for quality measured by M-NOS (Tables 1A,B provides more information).

**TABLE 1A:** Characteristics of the included 12 studies.

Study	Country	Design	Patients	Sample size	Age (mean)	Sex (male, %)	NYHA class III, IV (%)	LVEF (%)	Quality score
Yokota et al. (12)	Japan	R	AHF^a^	224	81	46	88	48.8 ± 9.5	4
Kanda et al. (17)	Japan	R	ADHF^b^	2,985	77.7	53.5	88.65	NA	5
Van Nguyen et al. (7)	Vietnam	P	CHF	180	80.6	50	85	41.9 ± 10.2	3
Katano et al. (8)	Japan	R	HF^d^	413	78	50	36	48.3 ± 16.1	4
Manemann et al. (13)	US	P	HF^d^	2,692	73.6	46.3	NA	NA	4
Chivite et al. (18)	Spain	P	AHF^a^	2,195	83	57	NA	< 50%:35.0%	5
Murad et al. (14)	US	P	HF^c^	558	79.2	48.2	42.4	≥ 45%: 48.3%*	4
Skalska et al. (11)	Poland	C	HF^d^	4,735	73.8	37.9	NA	NA	5
Rodríguez-Pascual et al. (19)	Spain	P	ADHF^b^	581	85.8	33	NA	< 45%: 24.7%	3
Wong et al. (10)	US	C	HF^d^	441	68.4	54.4	NA	NA	4
Gure et al. (15)	US	C	CHF	400	77.6	42.2	NA	NA	4
Vaccarino et al. (16)	US	P	HF^d^	391	NA	50.6	NA	< 40%:51.4%	4

*R, retrospective cohort study. P, prospective cohort study. C, cross-sectional study. AHF, acute heart failure. ADHF, acute decompensated heart failure. CHF, chronic heart failure. HF, heart failure.*

*^a^De novo cases or decompensations of a previously known HF.*

*^b^Decompensations of a previously known HF.*

*^c^De novo cases.*

*^d^De novo cases or previously known HF. NA, not available. NYHA, New York Heart Association. LVEF, left ventricular ejection fraction.*

*The percentage of LVEF ≥ 45% is on the basis of only 294 participants with known left ventricular ejection fraction in this study.

**TABLE 1B:** ADL-related characteristics of the 12 included studies.

Study	Assessment scale	Assessment time (Sources)	ADL impairment	
			Cut-off	Prevalence
Yokota et al. (12)	BI	Admission (Physical therapist)	<85	0.705
Kanda et al. (17)	BI	Admission (Self-report)	<60	0.629
Van Nguyen et al. (7)	KI	Discharge (Self-report)	≤5	0.261 HFpEF:0.234
Katano et al. (8)	BI	Discharge (Physical therapist)	<85	0.412 HFrEF:0.300
Manemann et al. (13)	Bathing, getting in and out of bed, feeding themselves, dressing, using the toilet, preparing meals, managing medications, and using transportation. ^a^	Out-of-hospital (Self-report)	>8	0.403
Chivite et al. (18)	BI	Out-of-hospital (Self-report)	≤60	0.217 HFrEF + HFmrEF:0.350
Murad et al. (14)	Walking around the home, getting out of bed, eating, dressing, bathing, and using the toilet. ^b^	Out-of-hospital (Self-report)	0 ADL impaired 1 ADL impaired ≥ 2 ADL impaired	0.775 0.125 0.100 0.225*
Skalska et al. (11)	KI	Out-of-hospital (Self-report)	6 ≤5 ≤2	0.737 0.263* 0.090
Rodríguez-Pascual et al. (19)	KI	Out-of-hospital (Self-report)	6 4–5 ≤3	0.327 0.281 0.391 0.672*
Wong et al. (10)	Dressing, eating, getting in and out of bed.^b^	Out-of-hospital (Self-report)	At least one ADL can’t be done independently	0.111
Gure et al. (15)	Bathing, dressing, eating, toileting, walking and transferring.^b^	Out-of-hospital (Self-report)	0 ADL impaired 1–3 ADL impaired 4–6 ADL impaired	0.457 0.376 0.168 0.544*
Vaccarino et al. (16)	KI	Out-of-hospital (Self-report)	6 5 ≤4	0.788 0.082 0.130 0.212*

*BI, The Barthel Index. BI consists of 10 items concerning functional capability regarding ADL (e.g., feeding, dressing, and chair/bed transfer). The score ranges from 0 (totally dependent when carrying out ADL) to 100 (fully independent in carrying out ADL); KI, The Katz Index. Participants were asked to identify whether they have any difficulty performing 6 items concerning functional capability regarding ADL (bathing, using the toilet, transferring, dressing, eating and continence.) on their own. The response options were binary (Yes = 1/No = 0), The score ranges from 0 (totally dependent) to 6 (fully independent).*

*^a^8-items ADL scale on a 5- point Likert scale (1 = without any difficulty to do,5 = unable to do, score range 8–40).*

*^b^Participants were asked to identify whether they have any difficulty performing these activities on their own. The response options were binary (Yes/No).*

*The prevalence of people who are unable to conduct at least one ADL independently; HFpEF, heart failure with preserved ejection fraction; HFrEF, heart failure with reduced ejection fraction; HFmrEF, heart failure with mid-range ejection fraction.

### Pooled Prevalence of Activities of Daily Living Impairment in Patients With Heart Failure

**FIGURE 2:**
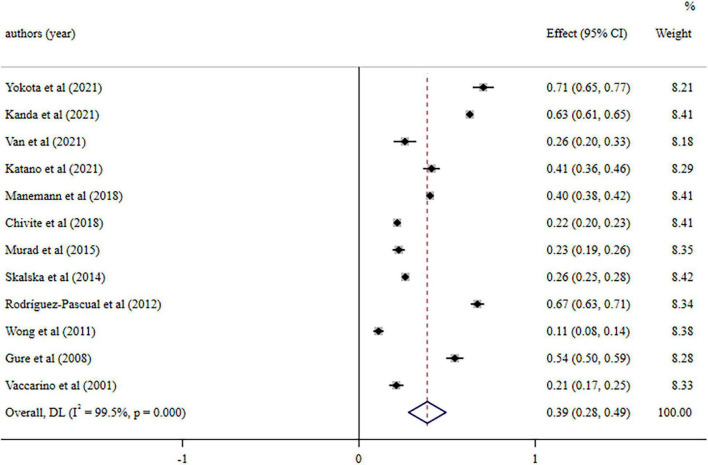
Forest plot of eligible studies. Weights are from random-effects model.

A total of 12 studies were included in the meta-analysis. The pooled prevalence of ADL impairment was 38.8% (95%CI: 28.2–49.3%; *I*^2^ = 99.5%, *P* < 0.001) (Figure 2).

### Subgroup Analysis

Subgroup analysis showed that the pooled prevalence of ADL impairment in Asian, Europe and America regions was 50.3% (95%CI: 33.2–67.3%), 38.3% (95%CI: 21.4–55.2%), and 30.0% (95%CI: 15.7–44.1%) (*Q* = 3.25, *P* = 0.197). In the subgroup analysis of assessment scale, the prevalence was 49.0% (95%CI: 23.1–75.0%) by using BI, 32.1% (95%CI: 15.1–49.2%) by use other scale, and 35.2% (95%CI: 15.4–55.1%) by using KI (*Q* = 1.17, *P* = 0.556). The prevalence of was 66.2% (95%CI: 58.7–73.6%) on admission. When discharged from the hospital, the prevalence was 33.8% (95%CI: 19.1–48.5%). When measured in the out-of-hospital setting, such as in a community setting or prior to hospital admission, the prevalence was 33.0% (95%CI: 23.6–42.5%) (*Q* = 35.07, *P* < 0.001). In the assessment method subgroup analysis, the prevalence was 55.8% (95%CI: 27.0–84.6%) when the assessment was made by physical therapist, higher than 35.4% (95%CI: 23.9–46.7%) by self-report (*Q* = 1.66, *P* = 0.197). However, none of the subgroups might be a source of heterogeneity (Table 2).

**TABLE 2:** Subgroup analysis of the pooled prevalence.

Subgroup	Studies	Pooled prevalence (95%CI)	*I* ^2^	Test of difference within each subgroup	
				Q	*P*
Region				3.25	0.197
Asia	4	0.50 (0.33, 0.67)	98.4%		
America	5	0.30 (0.16, 0.44)	99.0%		
Europe	3	0.38 (0.21, 0.55)	99.6%		
Country				18.57	0.001*
Japan	3	0.58 (0.43, 0.73)	97.5%		
Vietnam	1	0.26 (0.20, 0.33)	-		
Spain	2	0.44 (-0.00, 0.89)	99.8%		
Poland	1	0.26 (0.25, 0.27)	–		
US	5	0.30 (0.16, 0.44)	99.0%		
Assessment scale				1.17	0.556
BI	4	0.49 (0.23, 0.75)	99.7%		
KI	4	0.35 (0.15, 0.55)	99.3%		
Other	4	0.32 (0.15, 0.49)	99.2%		
Assessment time				35.07	< 0.001**
Admission	2	0.66 (0.59, 0.74)	82.8%		
Discharge	2	0.34 (0.19, 0.49)	92.7%		
Out-of-hospital	8	0.33 (0.24, 0.43)	99.2%		
Method of assessment				1.66	0.197
Physical therapist	2	0.56 (0.27, 0.85)	98.2%		
Self-report	10	0.35 (0.24, 0.47)	99.6%		
Sample size				0.01	0.906
≥ 2,000	4	0.38 (0.20, 0.55)	99.8%		
< 2,000	8	0.39 (0.23, 0.55)	99.2%		
Design				2.44	0.296
Prospective cohort	7	0.38 (0.26, 0.51)	99.2%		
Retrospective cohort	2	0.52 (0.31, 0.73)	98.6%		
Cross-sectional	3	0.31 (0.13, 0.48)	99.1%		
Quality score				0.20	0.904
3	2	0.47 (0.06, 0.87)	99.1%		
4	7	0.37 (0.24, 0.50)	99.0%		
5	3	0.37 (0.13, 0.61)	99.9%		
Publication time				0.84	0.359
<2017	6	0.34 (0.20, 0.48)	99.3%		
2017–2021	6	0.44 (0.28, 0.60)	99.6%		

*P < 0.05, **P < 0.001. BI, The Barthel Index. KI, The Katz Index.

### Meta-Regression Analysis

Meta-regression was performed on region, country, assessment scale, assessment time, method of assessment, sample size, study design, quality score, and publication time. The result indicated that assessment time accounted for 25.64% of the overall heterogeneity (*B* = -0.1450, *P* = 0.055) (Table 3).

**TABLE 3:** Meta-regression analyses of the effects of potential moderators.

Variables	B	95% Confidence interval		Adjusted *R*^2^	*P*-value
		Lower	Upper		
Region	–0.0678	–0.2429	0.1073	−2.32%	0.408
Country	–0.0239	–0.1192	0.0714	−6.79%	0.589
Assessment scale	–0.0846	0.2424	0.0732	3.72%	0.260
Assessment time	–0.1450	–0.2938	0.0038	25.64%	0.055
Method of assessment	–0.2039	–0.5461	0.1382	6.44%	0.214
Sample size	–0.0143	–0.3059	0.2772	−10.03%	0.915
Design	–0.0263	–0.1873	0.1347	−8.65%	0.723
Quality score	–0.0435	–0.2565	0.1695	−7.82%	0.659
Publication time	–0.1002	–0.3665	0.1662	−2.77%	0.422

### Sensitivity Analysis and Publication Bias

**FIGURE 3:**
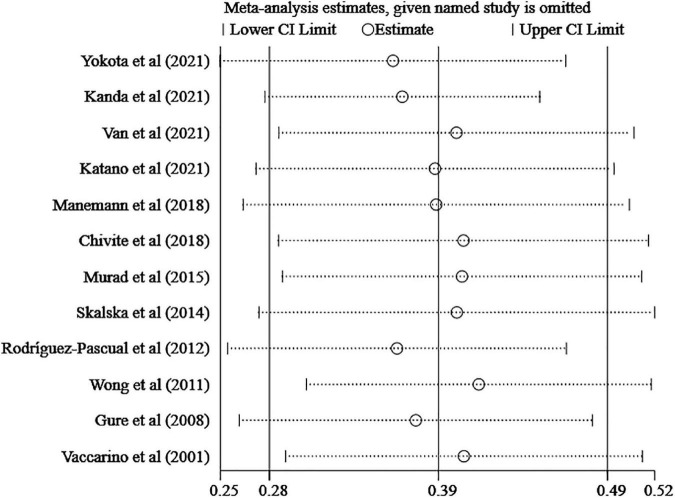
Sensitivity analysis estimating heterogeneity.

**FIGURE 4:**
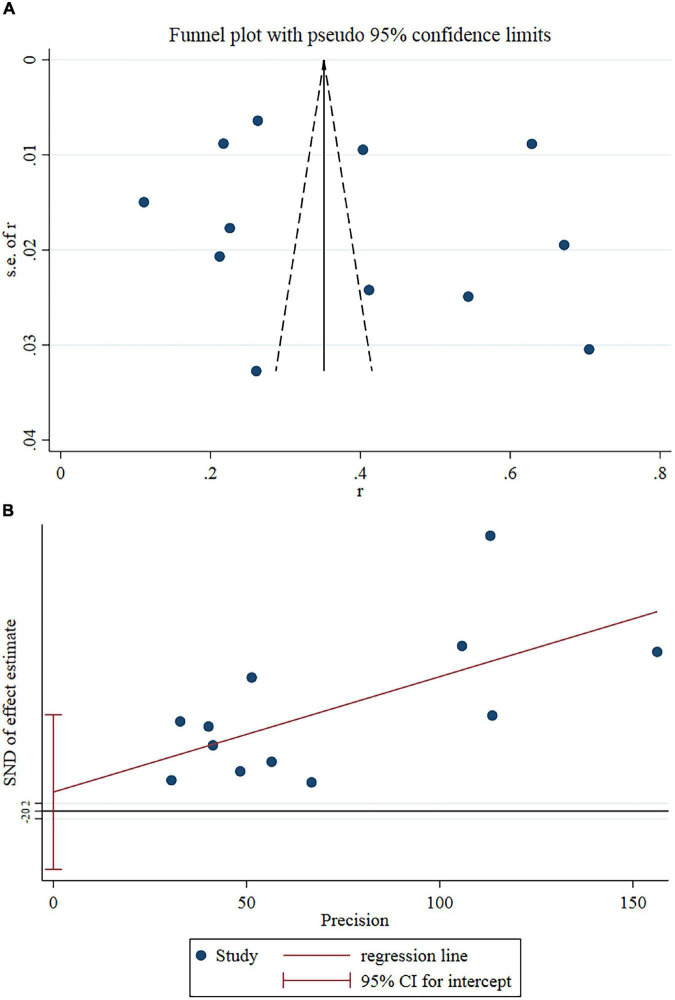
**(A)** Funnel plots of eligible studies showed slight asymmetry. **(B)** Egger’s test showed no publication bias.

Sensitivity analysis revealed no outlier studies that might significantly alter the primary results (Figure 3). Although the funnel plot showed slight asymmetry (Figure 4A), Egger’s test did not support publication bias (*t* = 0.55, *P* = 0.595) (Figure 4B).

## Discussion

This systematic review and meta-analysis included 12 studies with a total sample size of 15,795 HF patients. The pooled results showed that 38.8% of HF patients suffer from ADL impairment. As far as we know, this meta-analysis is the first to quantitative the prevalence of ADL impairment in patients with HF worldwide.

The prevalence of ADL impairment in HF patients had a significant difference between countries (*P* = 0.001). The prevalence of ADL impairment in Asian was 50.3%, which was the highest among the three regions. In our review, the highest prevalence of ADL impairment in Japanese HF patients was 58.2%. Firstly, Japan is the country with the fastest aging population in the world. The number of HF patients in Japan, especially the elderly, is increasing rapidly (20). Secondly, this may be attributed to cultural differences (21). Many Asians have followed a culture of stoicism, especially in Japan. Patients tend to conceal their distressing symptoms in Asian cultures. Thirdly, the disparities in medical systems between countries also may explain the different prevalence of ADL impairment. Some reviews indicated that patients with HF in developing countries such as the Philippines and Vietnam receive lower-than-recommended levels of pharmacological therapy. They may experience more severe signs or symptoms than patients in developed countries (22, 23). As a cost-effective medical intervention, the variation in cardiac rehabilitation levels may also result in the disparity in the frequency of ADL impairment between different countries (24). Cardiac rehabilitation has been shown in a rising number of studies to enhance physical function and the ability to conduct everyday tasks in HF patients (25, 26). It is a pity that cardiac rehabilitation is available in only 17% of countries in Southeast Asia (27). The participation rate of cardiac rehabilitation remains low in developing countries, which may be related to the awareness of cardiac rehabilitation among patients and doctors, patients’ economic level, health insurance coverage, and the distance between patients’ residence and rehabilitation centers (28, 29).

The prevalence of ADL impairment in America and Europe regions was 30.0 and 38.3%. In Western cultures, patients are more likely to maintain a positive attitude toward the act of seeking medical care (30). Patients with HF in developed countries have nationalized health care and easy access to cardiologists. A global cross-sectional survey showed that cardiac rehabilitation services are available in 80.7% of European countries and 70.0% of American countries. All of the reasons above may explain the differences in ADL impairment between countries. But this review lack of enough studies to support the effect of the level of cardiac rehabilitation development on ADL impairment in HF patients. Thus, we suggest that more nations with insufficient levels of cardiac rehabilitation improve ADL in HF patients through cardiac rehabilitation.

Significant differences in the prevalence of ADL impairment may be related to assessment time. The highest prevalence of impaired ADL in HF patients was at admission (66.2%), followed by discharge (33.8%) and lowest in the out-of-hospital setting (33.0%). The higher ADL impairment prevalence in patients with HF in the acute setting (e.g., on admission) is reasonable. It can be explained by the presence of dyspnea, chest pain, and edema in the decompensated phase of HF patients (5). The HF symptoms described above objectively limit the ability of HF patients to perform daily activities. Some patients yield to avoid suffering the painful experience of HF symptoms and develop a fear of daily activities, which subjectively leads to impaired ADL. The prevalence at discharge (33.8%) was lower than that on admission (66.2%). After a series of medications, care and cardiac rehabilitation during hospitalization, it is reasonable for HF patients to have some recovery in their ability to perform daily living compared to the time of admission (26). B-type natriuretic peptide (BNP) is a cardiac hormone produced and secreted by the heart. Plasma BNP levels increase in proportion to the severity of heart failure and they decrease as treatment improves the patient’s condition (31). As changes in BNP level reflect hemodynamic deterioration/improvement, BNP reduction during hospitalization has the potential to add ADL improvement (32). The prevalence in out-of-hospital setting (such as community settings or prior to hospitalization) was 33.0%, lower than at admission or at discharge. Currently, impaired ADL in the out-of-hospital setting have received little attention. But still, we may not ignore the huge number of HF patients in the community. After all, hospitalized HF patients only account for about one-third of the total number of HF patients (33). Therefore, ADL impairment in HF patients should be taken into consideration by community health workers. Meanwhile, there are few longitudinal studies on ADL impairment in HF patients with different disease periods. We recommend that future studies focus on changes in ADL ability in patients with HF, so that appropriate interventions can be developed accordingly to enhance ADL ability.

However, the present study also has the following limitations. First, more than half of the included studies were conducted in Europe and the US, and for some reason there is a lack of relevant studies on ADL impairment in HF patients in other countries. This made the consult potentially unrepresentative on global level. Considering the imbalance of health care resources in different regions, we suggest that more studies should be conducted in different regions to understand the overall situation. Second, due to limited data from the original study, quantitative assessment of ADL impairment was lacking, as well as subgroup analysis of some influencing factors associated with ADL impairment, such as LVEF, BMI, and age, was not performed. It is expected that future studies will provide more original data to explore the sources of inter-study heterogeneity from the above perspectives. Last, the assessment tool of ADL included in this review were based on subjective feedback of the patients, which may not be objective enough. We expect that future studies will use more objective tools to evaluate ADLs (e.g., accelerometers).

## Conclusion

The prevalence of ADL impairment in HF patients was 38.8%. Additionally, differences in the prevalence of ADL impairment in patients with HF may stem from country, region, and assessment time. We suggest that more researchers focus on the changes of ADL impairment in HF patients during different disease periods in different regions and countries.

## Data Availability Statement

The original contributions presented in the study are included in the article/Supplementary Material, further inquiries can be directed to the corresponding author/s.

## Author Contributions

JL and YZ searched and checked the databases according to the inclusion and exclusion criteria, extracted the data, and assessed their quality. JL analyzed the data and wrote the draft of the manuscript. RY and MW gave advice on meta-analysis methodology and revised the manuscript. XS was the guarantors of this work and had full access to all the data in the study and took responsibility for its integrity and the accuracy of the data analysis. All authors contributed to reviewing read and approved the final manuscript.

## Conflict of Interest

The authors declare that the research was conducted in the absence of any commercial or financial relationships that could be construed as a potential conflict of interest.

## Publisher’s Note

All claims expressed in this article are solely those of the authors and do not necessarily represent those of their affiliated organizations, or those of the publisher, the editors and the reviewers. Any product that may be evaluated in this article, or claim that may be made by its manufacturer, is not guaranteed or endorsed by the publisher.
