# Supplementary material for: The Prevalence of Activities of Daily Living Impairment in Patients With Heart Failure: A Systematic Review and Meta-Analysis
Source: Front Cardiovasc Med. 2022 Jul 14;9:952926. doi: 10.3389/fcvm.2022.952926 (PMC9330145; doi:10.3389/fcvm.2022.952926)
Supplement: Supplementary file 1 [file Table_1.DOCX]

**Supplementary file 1：**Search strategy

**Part 1. English databases**

**1. Pubmed**

| **Search number** | **Query** |
| --- | --- |
| 1 | "Heart Failure"[MeSH Terms] |
| 2 | "heart failure"[Title/Abstract] OR "cardiac failure"[Title/Abstract] OR "cardiac dysfunction"[Title/Abstract] OR "cardiac insufficiency"[Title/Abstract] OR "myocardial failure"[Title/Abstract] OR "heart decompensation"[Title/Abstract] OR "congestive heart failure"[Title/Abstract] |
| 3 | "Heart Failure"[MeSH Terms] OR "Heart Failure"[Title/Abstract] OR "cardiac failure"[Title/Abstract] OR "cardiac dysfunction"[Title/Abstract] OR "cardiac insufficiency"[Title/Abstract] OR "myocardial failure"[Title/Abstract] OR "heart decompensation"[Title/Abstract] OR "congestive heart failure"[Title/Abstract] |
| 4 | "Activities of Daily Living"[MeSH Terms] |
| 5 | "activities of daily living"[Title/Abstract] OR "ADL"[Title/Abstract] OR "activities daily living"[Title/Abstract] OR "activity daily living"[Title/Abstract] OR "daily living activities"[Title/Abstract] OR "daily living activity"[Title/Abstract] OR "living activities daily"[Title/Abstract] OR (("lived"[All Fields] OR "lives"[All Fields] OR "Living"[All Fields] OR "livings"[All Fields]) AND "activity daily"[Title/Abstract]) OR "chronic limitation of activity"[Title/Abstract] |
| 6 | "Activities of Daily Living"[MeSH Terms] OR ("Activities of Daily Living"[Title/Abstract] OR "ADL"[Title/Abstract] OR "activities daily living"[Title/Abstract] OR "activity daily living"[Title/Abstract] OR "daily living activities"[Title/Abstract] OR "daily living activity"[Title/Abstract] OR "living activities daily"[Title/Abstract] OR (("lived"[All Fields] OR "lives"[All Fields] OR "Living"[All Fields] OR "livings"[All Fields]) AND "activity daily"[Title/Abstract]) OR "chronic limitation of activity"[Title/Abstract]) |
| 7 | ("Heart Failure"[MeSH Terms] OR ("Heart Failure"[Title/Abstract] OR "cardiac failure"[Title/Abstract] OR "cardiac dysfunction"[Title/Abstract] OR "cardiac insufficiency"[Title/Abstract] OR "myocardial failure"[Title/Abstract] OR "heart decompensation"[Title/Abstract] OR "congestive heart failure"[Title/Abstract])) AND ("Activities of Daily Living"[MeSH Terms] OR ("Activities of Daily Living"[Title/Abstract] OR "ADL"[Title/Abstract] OR "activities daily living"[Title/Abstract] OR "activity daily living"[Title/Abstract] OR "daily living activities"[Title/Abstract] OR "daily living activity"[Title/Abstract] OR "living activities daily"[Title/Abstract] OR (("lived"[All Fields] OR "lives"[All Fields] OR "Living"[All Fields] OR "livings"[All Fields]) AND "activity daily"[Title/Abstract]) OR "chronic limitation of activity"[Title/Abstract])) |

**2. Cochrane Library**

| **ID** | **Search** |
| --- | --- |
| #1 | MeSH descriptor: [Heart Failure] explode all trees |
| #2 | (heart failure):ti,ab,kw |
| #3 | (cardiac failure):ti,ab,kw |
| #4 | (cardiac dysfunction):ti,ab,kw |
| #5 | (cardiac insufficiency):ti,ab,kw |
| #6 | (Myocardial failure):ti,ab,kw |
| #7 | (Heart Decompensation):ti,ab,kw |
| #8 | (Congestive Heart Failure):ti,ab,kw |
| #9 | #1 OR #2 OR #3 OR #4 OR #5 OR #6 OR #7 OR #8 |
| #10 | MeSH descriptor: [Activities of Daily Living] explode all trees |
| #11 | (Activities of Daily Living):ti,ab,kw |
| #12 | (ADL):ti,ab,kw |
| #13 | (Activities, Daily Living):ti,ab,kw |
| #14 | (Activity, Daily Living):ti,ab,kw |
| #15 | (Daily Living Activities):ti,ab,kw |
| #16 | (Daily Living Activity):ti,ab,kw |
| #17 | (Living Activities, Daily):ti,ab,kw |
| #18 | (Living Activity, Daily):ti,ab,kw |
| #19 | (Limitation of Activity, Chronic):ti,ab,kw |
| #20 | (Chronic Limitation of Activity):ti,ab,kw |
| #21 | (adl disability):ti,ab,kw |
| #22 | #10 OR #11 OR #12 OR #13 OR #14 OR #15 OR #16 OR #16 OR #17 OR #18 OR #19 OR #20 #21 |
| #23 | #9 AND #22 |

**3. Embase**

| **No.** | **Query** |
| --- | --- |
| #1 | 'heart failure'/exp |
| #2 | 'heart failure':ti,ab |
| #3 | 'cardiac failure':ti,ab |
| #4 | 'cardiac dysfunction':ti,ab |
| #5 | 'cardiac insufficiency':ti,ab |
| #6 | 'myocardial failure':ti,ab |
| #7 | 'heart decompensation':ti,ab |
| #8 | 'congestive heart failure':ti,ab |
| #9 | #1 OR #2 OR #3 OR #4 OR #5 OR #6 OR #7 OR #8 |
| #10 | 'daily life activity'/exp |
| #11 | 'adl disability'/exp |
| #12 | 'activities of daily living':ab,ti |
| #13 | 'adl':ab,ti |
| #14 | 'activities, daily living':ab,ti |
| #15 | 'activity, daily living':ab,ti |
| #16 | 'daily living activities':ab,ti |
| #17 | 'daily living activity':ab,ti |
| #18 | 'living activities, daily':ab,ti |
| #19 | 'living activity, daily':ab,ti |
| #20 | 'limitation of activity, chronic':ab,ti |
| #21 | 'chronic limitation of activity':ab,ti |
| #22 | 'adl disability':ab,ti |
| #23 | #10 OR #11 OR #12 OR #13 OR #14 OR #15 OR #16 OR #17 OR #18 OR #19 OR #20 OR #21 OR #22 |
| #24 | #9 AND #23 |

**4. CINAHL**

| **#** | **Query** |
| --- | --- |
| S1 | MH heart failure |
| S2 | TI heart failure OR AB heart failure |
| S3 | TI cardiac failure OR AB cardiac failure |
| S4 | TI cardiac dysfunction OR AB cardiac dysfunction |
| S5 | TI Myocardial failure OR AB Myocardial failure |
| S6 | TI Heart Decompensation OR AB Heart Decompensation |
| S7 | (S1 OR S2 OR S3 OR S4 OR S5 OR S6) |
| S8 | MH Activities of Daily Living |
| S9 | TI Activities of Daily Living OR AB Activities of Daily Living |
| S10 | TI ADL OR AB ADL |
| S11 | TI Activities, Daily Living OR AB Activities, Daily Living |
| S12 | TI Activity, Daily Living OR AB Activity, Daily Living |
| S13 | TI Daily Living Activities OR AB Daily Living Activities |
| S14 | TI Daily Living Activity OR AB Daily Living Activity |
| S15 | TI Living Activities, Daily OR AB Living Activities, Daily |
| S16 | TI Living Activity, Daily OR AB Living Activity, Daily |
| S17 | TI Limitation of Activity, Chronic OR AB Limitation of Activity, Chronic |
| S18 | (S8 OR S9 OR S10 OR S11 OR S12 OR S13 OR S14 OR S15 OR S16 OR S17) |

**Part 2. Chinese databases**

**CNKI / Wanfang / SinoMed/VIP:**

("heart failure" OR "cardiac failure" OR "cardiac dysfunction" OR "cardiac insufficiency" OR "myocardial failure" OR "heart decompensation" OR "congestive heart failure") AND ("activities of daily living")
